# Early Versus Late Ileostomy Closure After Anterior Rectal Resection: Association With Postoperative Complications

**DOI:** 10.7759/cureus.102688

**Published:** 2026-01-31

**Authors:** Victor Viegas, Hugo Pais Moreira, Daniel Martins, Bruno Calisto, Ana Ferreira, Tatiana Queirós, Miguel Mendes, João Miguel Cardoso, Lurdes Gandra, Bela Pereira

**Affiliations:** 1 General Surgery, Unidade Local de Saúde Gaia e Espinho, Vila Nova de Gaia, PRT

**Keywords:** anterior rectal resection, ileostomy closure, postoperative complications, protective ileostomy, rectal cancer

## Abstract

Background: Protective ileostomy is often performed following anterior resection for rectal cancer to minimize the clinical consequences of anastomotic leakage. The actual operation to create the stoma is usually straightforward, although ileostomy management can result in considerable patient morbidity. The timing of ileostomy closure continues to be a subject of debate. While early closure of the stoma may alleviate an individual’s experience of prolonged skin irritation or persistent concerns regarding hydration, it still raises concerns about postoperative safety.

Methods: We performed a retrospective, single-center study, analyzing patients who had anterior rectal resection (ARR) with a protective ileostomy between January 2016 and December 2023. From the electronic records, we collected clinical data, oncologic characteristics, operative notes, ileostomy closure data, and any postoperative complications (graded by Clavien-Dindo). For analysis, patients were divided into two groups: early closure (≤30 days) and late closure (>30 days). A multivariable model was used to evaluate factors associated with postoperative complications.

Results: Our cohort included 85 patients, with a mean age of 61.9 ± 11.1 years and a male predominance. The timing of ileostomy closure varied across the patients, with a median of 60 days. Around 24 patients (28.2%) had early closure, while 61 patients (71.8%) underwent late closure. The median operative time was 64.5 minutes, with a median post-operative hospital stay of six days. Complications occurred in 26 patients (30.6%), and 84.6% (22 patients) were Clavien-Dindo grade II. Surgical site infections (SSIs) were the most frequent complication. On multivariable analysis, early ileostomy closure (p = <0.001) was significantly associated with a higher rate of postoperative complications.

Conclusions: In our study, early closure of protective ileostomy was found to be associated with an increase in postoperative complications, mainly minor complications (Clavien-Dindo grade II). Although early reversal may offer benefits in reducing stoma-related problems, our findings highlight the importance of individualized patient selection, considering patient comorbidities, perioperative risk factors, and clinical context in order to optimize patient outcomes.

## Introduction

Anterior rectal resection (ARR) remains a cornerstone surgery for rectal cancer [1] and, despite refinements in surgical technique, perioperative care, and assessment of anastomotic integrity, the procedure still carries an important risk of anastomotic leakage, ranging from 3% to 23% [2,3].

When an anastomotic leakage occurs, it can carry serious consequences. Patients often face considerable morbidity, and hospital stays are typically extended well beyond the expected course. Mortality risk is not negligible, and there is concern that such complications may also compromise long‑term oncologic outcomes [4,5].

Protective ileostomy is often used to help minimize the clinical implications associated with anastomotic leakage. By limiting the amount of stool passing over the new anastomosis, diversion can lessen the physiological strain on the site and, if a leak develops, may reduce its clinical severity [6,7]. Much evidence supports this strategy, showing that diverting stomas are effective in decreasing the consequences of anastomotic complications [8,9].

Despite the benefits of a protective ileostomy, patients may face a variety of stoma‑related morbidities that can meaningfully reduce quality of life and daily functioning [10]. These tend to be practical and sometimes persistent: peristomal skin irritation, electrolyte disturbances that may require repeated blood tests and corrections, episodes of dehydration severe enough to prompt readmission, and the later development of parastomal hernia; alongside these physical issues, many patients report anxiety, body-image concerns, and social withdrawal that interfere with work, travel, or simple daily activities [11,12]. These factors may lead patients and surgeons to consider the possibility of an earlier closure after a careful assessment of the risks and benefits.

Strong views on both early and delayed approaches exist in the literature, and the timing of ileostomy closure is still a matter of debate [13-15]. Those in favor of early closure argue that it may shorten the period of stoma-related problems, help patients return to normal routines sooner, and ease the demands of ongoing stoma care [13].

Because the evidence around the commonly used 30‑day cutoff is limited, we carried out a retrospective study to evaluate the association between early (≤30 days) versus late (>30 days) ileostomy closure and postoperative complications following ARR. Our goal was to provide practical data that may guide everyday decisions about when to reverse a temporary ileostomy.

## Materials and methods

Study design and setting

We conducted a retrospective, single-center cohort study at the Department of Surgery, Unidade Local de Saúde Gaia e Espinho, Vila Nova de Gaia, Portugal. The study concentrated on patients with rectal cancer who underwent ARR with a protective ileostomy, with the intention of examining whether the timing of ileostomy closure might be linked to postoperative complications. This retrospective study was conducted in accordance with institutional policies governing research using anonymized clinical data. Under these policies, studies involving the secondary use of existing data, with no direct patient contact and no collection of identifiable information, are exempt from formal ethics committee review and informed consent requirements. The study was conducted in compliance with applicable national regulations and with the principles of the Declaration of Helsinki.

Patient population

We reviewed all consecutive patients who underwent ARR with the construction of a protective ileostomy between January 2016 and December 2023. To be included, individuals had to meet several criteria: they were at least 18 years of age or older, had rectal cancer treated with ARR, received a protective ileostomy, and ultimately underwent ileostomy closure. Exclusion criteria were applied to patients whose ileostomy was never reversed, if medical records were incomplete, or if the stoma had been created in the context of another type of surgery. Patients who did not undergo ileostomy closure during the study period were excluded, as postoperative outcomes after closure constituted the primary endpoint of the study.

The timing of ileostomy closure was determined by the treating surgical team based on individualized clinical judgment rather than a standardized institutional protocol. Earlier closure was generally considered in patients who had an uncomplicated postoperative course after ARR, no clinical or radiological evidence of anastomotic leakage (confirmed by contrast imaging or endoscopic evaluation), adequate nutritional and functional status, and acceptable control of comorbidities. In addition, stoma-related morbidity, such as recurrent dehydration, electrolyte disturbances, frequent peristomal skin complications, difficulties with stoma management, or a significant impact on quality of life, often prompted consideration of earlier reversal. Conversely, delayed closure was more commonly chosen in patients with postoperative complications after the index surgery, delayed recovery, ongoing or planned adjuvant oncologic therapy, or concerns regarding anastomotic integrity.

Data collection and variables

Clinical data were systematically extracted from electronic medical records. Collected variables included patient demographics (age, gender, and body mass index (BMI)), American Society of Anesthesiologists (ASA) classification, and comorbidities (hypertension, diabetes mellitus, smoking status, dyslipidemia, cardiovascular disease, respiratory disease, and chronic renal disease).

Oncologic variables included tumor location, stage, and neoadjuvant and adjuvant therapy. Variables related to the rectal resection included surgical approach (laparoscopic versus laparotomic), surgical setting (elective or urgent), type of mesorectal excision (total or partial), and early postoperative complications.

Variables related to the ileostomy included the indication of its creation, time interval to ileostomy closure, operative time of ileostomy closure, length of hospitalization following reversal, and postoperative complications following reversal.

Study groups and definitions

In order to conduct our analysis, the patient population was divided into two groups according to the timing of ileostomy closure: those who underwent early closure, defined as ≤30 days of the index operation, and those whose closure occurred later, >30 days of the index operation. The 30-day cutoff was selected as a pragmatic definition of early ileostomy closure. Although no international guideline mandates a specific threshold, prior studies have frequently defined early closure as occurring within four weeks of the index operation [16].

We used the Clavien-Dindo system to classify any adverse event after ileostomy closure. The Clavien-Dindo classification is a standardized system that grades postoperative surgical complications according to the type and intensity of treatment required to manage them, ranging from mild deviations in recovery to death. It runs from Grade I, defined as any deviation from the normal postoperative course not needing specific pharmacologic, surgical, endoscopic, or radiologic intervention, through Grade II, which requires pharmacologic treatment such as antibiotics, blood transfusions, or parenteral nutrition. Grades IIIa and IIIb involve complications requiring surgical, endoscopic, or radiologic intervention without (IIIa) or with (IIIb) general anesthesia, respectively. Grade IV is reserved for life‑threatening complications requiring intensive care management, with IVa indicating single-organ dysfunction and IVb multi-organ dysfunction, while Grade V denotes the death of the patient [17]. Major complications were defined as grade III or higher.

Surgical site infection (SSI) was defined according to the Centers for Disease Control and Prevention criteria and classified as superficial incisional, deep incisional, or organ-space infection. SSIs were identified based on objective clinical findings (such as purulent drainage from the incision or organ space) and/or microbiological confirmation from culture-positive samples. All postoperative complications, including SSI, were recorded within 30 days following ileostomy closure.

Statistical analysis

Normally distributed variables are presented as mean ± standard deviation and compared using Student’s t-test. Non-normally distributed variables are presented as median (IQR) and compared using the Mann-Whitney U test. Categorical variables are presented as counts and percentages and compared using Fisher’s exact test.

A predictive model to assess the association and magnitude between clinical factors and the development of postoperative complications was constructed using logistic regression, including univariate and multivariable analyses. Given the limited number of postoperative complications, multivariable model construction prioritized parsimony and clinical relevance to minimize overfitting. Early ileostomy closure was included as the primary exposure of interest, and adjustment was limited to one pre-specified baseline risk covariate (age), selected a priori based on its established association with postoperative morbidity. Variables with low prevalence were not included in the final multivariable model. For all statistical tests, a two-sided p-value <0.05 was considered statistically significant. Analyses were performed using IBM SPSS Statistics for Windows, Version 29 (Released 2022; IBM Corp., Armonk, New York, United States).

The multivariable analysis was intended to identify factors statistically associated with postoperative complications and was not designed to establish causal relationships.

## Results

**Figure 1:**
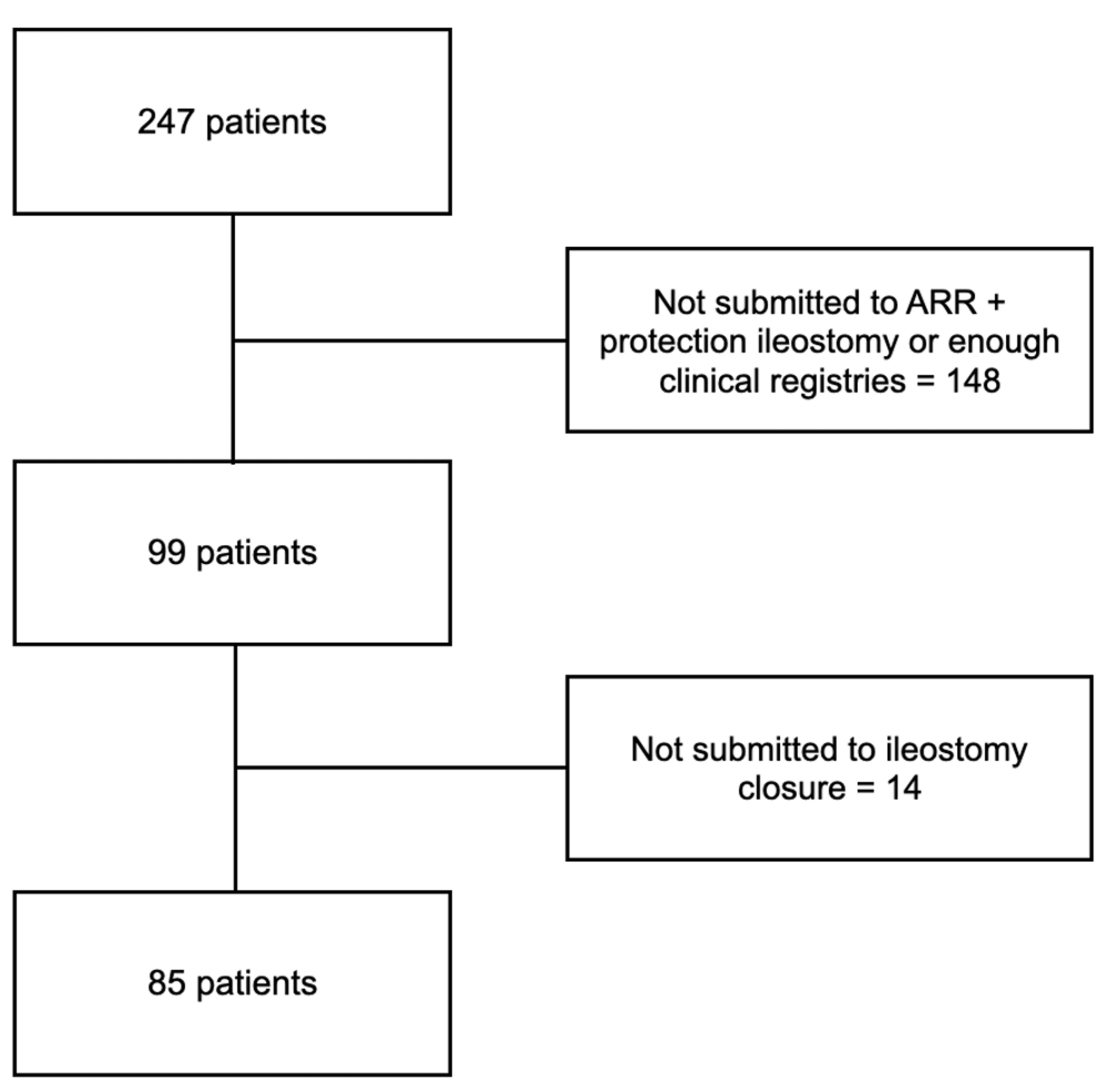
Flow diagram of patient selection for inclusion (January 2016 - December 2023). ARR: anterior rectal resection

From January 2016 to December 2023, 247 patients underwent surgery for rectal cancer at our institution. Among them, 99 (40.1%) received an ARR with a protective ileostomy. Once the inclusion and exclusion criteria were applied, 85 patients remained eligible, representing 34.4% of the original rectal surgery cohort. These individuals ultimately proceeded to ileostomy closure, forming the basis of our final analysis (Figure 1).

In our cohort, 14 patients with a protective ileostomy did not undergo reversal during the study period. The reasons recorded were heterogeneous: disease progression or recurrence, poor general condition or comorbidities that made reoperation unsafe, anastomotic complications, ongoing oncologic treatment, and, in some cases, patient preference. Those who remained with a stoma tended to have less favorable baseline profiles and more eventful early courses. Specifically, they exhibited a higher overall comorbidity burden and experienced postoperative complications after the index operation more often than patients who went on to reversal.

Patient characteristics

The mean age of the cohort was 61.9 years (± 11.1), with men accounting for the majority (58 patients, 68.2%). Most individuals were classified as ASA II (62 patients, 72.9%), and the average BMI was 26.0 ± 3.9 kg/m². The most frequent conditions that we observed were: hypertension (37 patients, 43.5%), dyslipidemia (22 patients, 25.9%), a history of smoking (20 patients, 23.5%), and diabetes mellitus (18 patients, 21.2%). Cardiovascular disease (seven patients, 8.2%), respiratory disorders (four patients, 4.7%), and chronic renal disease (two patients, 2.4%) appeared to be less common in our cohort (Table 1).

**Table 1:** Patient demographic data. *Mean ± standard deviation ASA: American Society of Anesthesiologists

Variable	Value n (%)	Early vs late closure (*p*)
Age *	61.94 ± 11.08	0.358
Gender	-	0.205
Male	58 (68.2%)	-
Female	27 (31.8%)	-
ASA	-	0.791
II	62 (72.9%)	-
III	23 (27.1%)	-
BMI *	26 ± 3.85	0.457
Comorbidities	-	-
Hypertension	37 (43.5%)	0.812
Dyslipidemia	22 (25.9%)	0.591
Smoking history	20 (23.5%)	0.087
Diabetes	18 (21.2%)	0.769
Cardiovascular disease	7 (8.2%)	1
Respiratory disease	4 (4.7%)	0.066
Chronic renal disease	2 (2.4%)	1

When comparing early and late closure groups, age, gender, ASA score, BMI, and all comorbidities did not show statistically significant differences. Smoking and respiratory disease were somewhat more frequent in the early closure group, though the differences did not reach statistical significance.

Oncologic and surgical characteristics

Neoadjuvant therapy was offered to 55 patients (64.7%). Within that group, 30 (35.3%) received chemoradiotherapy (CRT), 15 (17.6%) underwent total neoadjuvant therapy (TNT), and 10 (11.8%) had radiotherapy (RT) alone. Adjuvant chemotherapy was later administered to 35 patients (41.2%).

Stage IIIB was the most common (32 patients, 37.6%). There were 20 patients with stage I disease (23.5%) and 12 with stage IIIC disease (14.1%). Tumor location was more frequent in the mid‑rectum (47 patients, 55.3%), with smaller shares in the upper rectum (20 patients, 23.5%) and lower third (18 patients, 21.2%).

Most of the patients were submitted to laparoscopic surgery (62 patients, 72.9%); the remainder were laparotomic (23 patients, 27.1%). The surgeries were done largely in the elective setting (81 patients, 95.3%), and total mesorectal excision was done in 76 patients (89.4%).

Complications after ARR were noted in 33 patients (38.8%). The largest portion fell into Clavien-Dindo grade II (13 patients, 15.3%), while grades IIIa and IIIb each occurred in six patients (7.1%). Less common events were grade I (five patients, 5.9%) and grade IVa (three patients, 3.5%) (Table 2).

**Table 2:** Oncologic and surgical characteristics of the study cohort. CRT: chemoradiotherapy; TNT: total neoadjuvant therapy; RT: radiotherapy

Variable	Value n (%)
Neoadjuvant therapy	-
CRT	30 (35.3%)
TNT	15 (17.6%)
RT	10 (11.8%)
Adjuvant therapy	35 (41.2%)
Tumor stage	-
I	20 (23.5%)
II	8 (9.4%)
IIIA	6 (7.1%)
IIIB	32 (37.6%)
IIIC	12 (14.1%)
IVA	6 (7.1%)
IVB	1 (1.2%)
Location within the rectum	-
Low	18 (21.2%)
Medium	47 (55.3%)
High	20 (23.5%)
Surgical approach	-
Laparoscopic	62 (72.9%)
Laparotomic	23 (27.1%)
Surgical setting (with ileostomy construction)	-
Elective	81 (95.3%)
Urgent	4 (4.7%)
Type of mesorectal excision	-
Total	76 (89.4%)
Partial	9 (10.6%)
Complication	33 (38.8%)
Clavien-Dindo	-
I	5 (5.9%)
II	13 (15.3%)
IIIa	6 (7.1%)
IIIb	6 (7.1%)
IVa	3 (3.5%)

Indications for protective ileostomy

The most common reason for the creation of an ileostomy was a low anastomosis (59 patients, 69.4%), followed by technical difficulties (22 patients, 25.8%), colorectal anastomotic dehiscence (two patients, 2.4%), hemoperitoneum (one patient, 1.2%), and peritonitis (one patient, 1.2%) (Table 3).

**Table 3:** Indications for creation of a protective ileostomy.

Variable	Value n (%)
Low anastomosis	59 (69.4%)
Technical difficulties	22 (25.8%)
Colorectal anastomosis dehiscence	2 (2.4%)
Hemoperitoneum	1 (1.2%)
Peritonitis	1 (1.2%)

Ileostomy closure data

The median duration until ileostomy closure occurred was 60 days (IQR 206), with 24 patients (28.2%) receiving an early closure (≤30 days) and 61 patients (71.8%) submitted to a late closure (>30 days). The median surgical duration for ileostomy closure was 64.5 minutes (IQR 25), and the median length of postoperative hospital stay was six days (IQR 5). When comparing early and late closure groups, duration of ileostomy closure and length of postoperative hospital stay did not show statistically significant differences (Table 4). Specific stoma-related complications prompting earlier closure were not consistently documented in a standardized manner and therefore could not be formally analyzed.

**Table 4:** Ileostomy closure timing, operative details, and postoperative complications. *Median (IQR); the bold values indicate statistically significant results (p < 0.05).

Variable	Value n (%)	Early vs late closure (*p*)
Timing of closure in days *	60 (206)	-
Early closure (≤30 days)	24 (28.2%)	-
Duration of the ileostomy closure in minutes *	64.5 (25)	0.277
Length of hospitalization in days *	6 (5)	0.157
Complication	26 (30.6%)	<0.001
Clavien-Dindo	-	-
II	22 (84.6%)	<0.001
IIIb	2 (7.7%)	0.487
IVa	1 (3.8%)	1
V	1 (3.8%)	1

Complications

Complications occurred in 26 patients (30.6%) overall, including 17/24 (70.8%) patients in the early closure group and 9/61 (14.8%) in the late closure group. In univariate analysis, early ileostomy closure was associated with a significantly higher risk of postoperative complications (OR 14.03, 95% CI 4.54-43.41, p <0.001) (Table 5).

**Table 5:** Univariate and multivariate analysis of factors associated with postoperative complications after ileostomy closure. *Variables included in the final model; the bold values indicate the variables included in the final multivariable model. The multivariable model was intentionally restricted to age and early closure to minimize overfitting, given the limited number of outcome events.

Variable	Univariate OR (95% CI)	p	Multivariate OR (95% CI)	p
Age	0.996 (0.995-1.038) *	0.840	1.009 (0.959-1.061)	0.714
Gender	1.392 (0.502-3.862)	0.525	-	-
ASA	2.212 (0.812-6.021)	0.120	-	-
BMI	1.023 (0.908-1.151)	0.713	-	-
Hypertension	1.166 (0.461-2.949)	0.746	-	-
Diabetes	1.175 (0.387-3.569)	0.776	-	-
Smoking status	2.310 (0.816-6.537)	0.115	-	-
Dyslipidemia	1.429 (0.512-3.987)	0.496	-	-
Cardiovascular disease	3.394 (0.702-16.413)	0.129	-	-
Respiratory disease	7.565 (0.748-76.531)	0.087	-	-
Chronic renal disease	2.320 (0.140-38.581)	0.557	-	-
Duration of the ileostomy closure surgery	1.002 (0.991-1.013)	0.739	-	-
Early closure (≤30 days)	14.032 (4.536-43.410) *	<0.001	14.449 (4.592-45.465)	<0.001

The majority were Clavien-Dindo grade II (22 patients, 84.6%), and this profile appeared more frequently in the early‑closure subgroup (p <0.001). SSI accounted for the majority of postoperative complications and was all classified as Clavien-Dindo grade II events. No statistically significant difference was observed in Clavien-Dindo grade ≥IIIb complications between the early and late closure groups; however, the number of major events was small, limiting statistical power.

Serious events were uncommon: two cases of grade IIIb (7.7%), one of grade IVa (3.8%), and one fatal complication (grade V, 3.8%) (Table 4). SSI was the most frequent complication, affecting 21 patients (80.8%). Other complications were less observed in our cohort: one intestinal subocclusion (3.8%), two small bowel obstructions (7.7%), one anastomotic dehiscence (3.8%), and one hemoperitoneum (3.8%).

Risk factors for postoperative complications after closure

In univariate analysis, early ileostomy closure was significantly associated with postoperative complications after ileostomy closure (OR 14.032, 95% CI 4.536-43.410, p <0.001). Age was not significantly associated with postoperative complications in univariate analysis (OR 0.996, 95% CI 0.995-1.038, p = 0.840).

Given the limited number of outcome events, a parsimonious multivariable model was constructed, including early closure as the primary exposure of interest and age as a pre-specified baseline covariate. In the adjusted analysis, early ileostomy closure remained independently associated with postoperative complications (OR 14.449, 95% CI 4.592-45.465, p <0.001), whereas age was not significantly associated with the outcome (OR 1.009, 95% CI 0.959-1.061, p = 0.714) (Table 5).

## Discussion

Our research reveals that an early closure of protective ileostomy (≤30 days) following ARR correlates with a greater incidence of postoperative complications when compared to a late closure. The main complications observed after closure included Clavien-Dindo grade II issues (such as SSI and one episode of subocclusion). However, our results showed that early closure did not reach statistical significance in a rise in Clavien-Dindo grade ≥IIIb complications, nor in mortality rates or the length of hospital stays. Because major complications were infrequent, this study was underpowered to detect clinically meaningful differences in severe morbidity between groups, and the absence of statistically significant differences should not be interpreted as evidence of equivalence or safety.

Although early closure was associated with an increased rate of minor complications, these events were generally managed conservatively. Taken together, these findings may suggest the need to balance short‑term, predominantly low‑grade morbidity against the potential benefits of earlier stoma reversal when individualizing decisions regarding ileostomy closure.

Comparison with existing literature

Our results mirror several recent series that examined how the timing of ileostomy closure relates to postoperative outcomes.

A multicenter randomized controlled trial showed evidence of the feasibility of early ileostomy reversal in selected patients. Danielsen et al. demonstrated that closure within eight to 13 days after ARR was both feasible and safe when applied to strictly selected patients who met specific clinical criteria. The research indicated that early closure resulted in a reduction of stoma-related complications, while no significant increase in major complications (Clavien-Dindo grade IIIa or above) was detected among the groups. The findings of this study suggest that for patients without clinical or radiological signs of anastomotic leakage, early ileostomy closure offers a clinically relevant advantage by reducing overall morbidity and improving patient outcomes, thereby supporting its feasibility and efficacy in clinical practice [18].

A recent meta-analysis suggested that in carefully selected patient populations, early closure (defined as ≤4 weeks) can be performed safely without a substantial increase in major morbidity. The research indicated a markedly reduced incidence of skin irritation within the early closure group. Despite the observation of an elevated rate of SSI, there was no corresponding rise in mortality rates or the length of hospital stays within the early closure cohort [16].

Conversely, one retrospective study revealed that the closure of ileostomy prior to 8.5 weeks elevated the likelihood of complications and prolonged hospital stays, suggesting that ileostomy closure should be deferred until after this timeframe to mitigate morbidity [15].

Definitions of “early” closure vary widely across the literature. Some series consider closure within eight to 13 days to be early, while others use windows of four weeks [16,18]. We chose a 30‑day cutoff, which may capture a somewhat different patient mix than studies using much shorter intervals. In addition, selection criteria are rarely identical between reports: randomized trials often apply strict inclusion rules that can exclude frailer or more complex patients, so their findings may not represent everyday clinical practice.

Clinical implications

Our series showed a predominance of Clavien-Dindo grade II complications, which may suggest that although early closure is associated with higher overall morbidity, most adverse events were moderate and could be managed with medical therapy. 

SSI was the most common complication, affecting a substantial share of patients and mirroring what others have reported after stoma reversal [16]. That observation underlines practical points: careful tissue handling in the operating room, timely and appropriate antibiotic prophylaxis, and vigilant wound care after discharge are all likely to matter, especially when closure is performed early.

Balancing benefits and risks

While our data show a higher rate of complications after early closure, that finding should be weighed against potential benefits for selected patients. Recent work links longer stoma duration to a greater risk of major low anterior resection syndrome (LARS), with an optimal cutoff for predicting major LARS reported at roughly 4.2 months [19]. In other words, delaying reversal without a clear reason may worsen long‑term bowel function for some patients.

Prolonged stoma use also brings its own burdens: peristomal skin breakdown and excoriation, stoma retraction or prolapse, intermittent electrolyte disturbances, and psychosocial burden from living with a stoma [20]. For patients who struggle with these problems, frequent appliance changes, recurrent dermatitis, or persistent social withdrawal, earlier closure can meaningfully improve day‑to‑day quality of life, even if it carries a higher short‑term complication rate.

The presence of a protective ileostomy is not a formal contraindication to commencing adjuvant therapy on schedule. Patients frequently receive chemotherapy while the stoma remains in place. Nevertheless, the presence of a stoma and its associated effects may potentially impede the optimal delivery of chemotherapy [21].

A Japanese study found that the presence of an ileostomy within 12 months after rectal resection did not affect the chemotherapy drug dose that patients could receive. While not affecting the dose, the study indicated that the presence of an ileostomy within 12 months after rectal resection could delay the initiation of chemotherapy. As a consequence, the initiation of chemotherapy can be delayed due to several factors related to postoperative and stoma-related complications that necessitate a pause or postponement in treatment [22].

That said, the balance is rarely simple. Optimizing outcomes, therefore, depends on careful, individualized selection. In our view, reasonable candidates for earlier reversal are those who had an uncomplicated immediate postoperative course, demonstrate adequate nutritional reserve without recent weight loss, show no clinical or radiological signs of anastomotic compromise, and lack major uncontrolled comorbidities. Shared decision-making is essential: clinicians and patients must weigh the risk of mostly manageable, short-term problems against the possibility of better long-term function and fewer stoma-related issues.

Future directions

Due to the fact that this is a single-center and retrospective study, our findings underscore the need for prospective, multicenter studies that evaluate a 30-day threshold for ileostomy closure. Ideally, these studies should use consistent patient-selection criteria, include validated quality-of-life instruments, and follow patients long enough to capture functional outcomes that matter to everyday life, bowel function, continence, and return to normal activities, along with short-term surgical endpoints. Such an approach would give clinicians firmer ground for shared decision-making.

Developing practical, evidence‑based algorithms or simple scoring tools to guide timing decisions may help clinicians select patients who will benefit from earlier closure while keeping low complication risk.

Finally, improving perioperative care pathways is likely to matter. Attention to nutritional optimization, tighter control of medical comorbidities, and more standardized surgical and wound-care techniques may reduce the complications we observed with early closure.

Study limitations

Our study has some limitations. That fact that this is a single-center, retrospective study, the results may not generalize to other settings. In addition, our small sample size may also reduce statistical power; smaller but clinically relevant differences between groups could have gone undetected.

We were unable to report long‑term functional outcomes or validated quality‑of‑life measures, which confines our conclusions largely to short‑term morbidity.

Although stoma-related morbidity likely influenced the decision to pursue earlier closure in a subset of patients, these factors were not systematically captured and could not be quantified in the analysis. As a result, we were unable to formally compare planned versus reactive early closure or to evaluate specific stoma-related indications as determinants of timing.

Because ileostomy closure timing was determined by clinical judgment rather than a predefined protocol, selection bias and confounding by indication are inherent limitations of this study. Factors such as stoma-related morbidity, patient preference, and recovery trajectory, some of which were not fully quantifiable, likely influenced both the timing of closure and postoperative outcomes. Although multivariable adjustment was performed, unmeasured factors influencing closure timing may have affected the observed associations. As such, causal inferences cannot be drawn from these findings.

## Conclusions

Our findings reinforce an individualized, patient‑centered approach to timing ileostomy closure after ARR. Early closure (≤30 days) was linked to a higher rate of mostly minor postoperative complications (predominantly Clavien-Dindo grade II), and SSI was the single most frequent problem.

Deciding the timing of an ileostomy closure should be a careful, case‑by‑case process. Clinicians ought to weigh objective factors, overall physiological reserve, nutritional status, evidence of an intact anastomosis on imaging, and oncologic treatment requirements, alongside more subjective but equally important considerations such as the patient’s experience with stoma care (frequent appliance changes, recurrent peristomal dermatitis), work or caregiving responsibilities, and personal preferences.
